# I Spy with My Little Eye: A Paediatric Visual Preferences Survey of 3D Printed Tablets

**DOI:** 10.3390/pharmaceutics12111100

**Published:** 2020-11-17

**Authors:** Patricija Januskaite, Xiaoyan Xu, Sejal R. Ranmal, Simon Gaisford, Abdul W. Basit, Catherine Tuleu, Alvaro Goyanes

**Affiliations:** 1 Department of Pharmaceutics, UCL School of Pharmacy, University College London, 29-39 Brunswick Square, London WC1N 1AX, UK; patricija.januskaite.18@ucl.ac.uk (P.J.); xiaoyan.xu.13@ucl.ac.uk (X.X.); sejal.ranmal@ucl.ac.uk (S.R.R.); s.gaisford@ucl.ac.uk (S.G.); 2 FabRx Ltd., 3 Romney Road, Ashford, Kent TN24 0RW, UK; 3 Departamento de Farmacología, Farmacia y Tecnología Farmacéutica, I + D Farma Group (GI-1645), Universidad de Santiago de Compostela, 15782 Santiago de Compostela, A Coruña, Spain

**Keywords:** three-dimensional printing, additive manufacturing, personalized pharmaceuticals, printlets, pediatrics, printing formulations and dosage forms, visual preference, 3D printed drug products, MEDIMAKER 3D printing technology

## Abstract

3D printing (3DP) in the pharmaceutical field is a disruptive technology that allows the preparation of personalised medicines at the point of dispensing. The paediatric population presents a variety of pharmaceutical formulation challenges such as dose flexibility, patient compliance, taste masking and the fear or difficulty to swallow tablets, all factors that could be overcome using the adaptable nature of 3DP. User acceptability studies of 3D printed formulations have been previously carried out in adults; however, feedback from children themselves is essential in establishing the quality target product profile towards the development of age-appropriate medicines. The aim of this study was to investigate the preference of children for different 3D printed tablets (Printlets™) as an important precursor to patient acceptability studies. Four different 3DP technologies; digital light processing (DLP), selective laser sintering (SLS), semi-solid extrusion (SSE) and fused deposition modeling (FDM) were used to prepare placebo printlets with similar physical attributes including size and shape. A single-site, two-part survey was completed with participants aged 4–11 years to determine their preference and opinions based on visual inspection of the printlets. A total of 368 participants completed an individual open questionnaire to visually select the best and worst printlet, and 310 participants completed further non-compulsory open questions to elaborate on their choices. Overall, the DLP printlets were the most visually appealing to the children (61.7%) followed by the SLS printlets (21.2%), and with both the FDM (5.4%) and SSE (11.7%) printlets receiving the lowest scores. However, after being informed that the SSE printlets were chewable, the majority of participants changed their selection and favoured this printlet, despite their original choice, in line with children’s preference towards chewable dosage forms. Participant age and sex displayed no significant differences in printlet selection. Printlet descriptions were grouped into four distinct categories; appearance, perceived taste, texture and familiarity, and were found to be equally important when creating a quality target product profile for paediatric 3D printed formulations. This study is the first to investigate children’s perceptions of printlets, and the findings aim to provide guidance for further development of paediatric-appropriate medicines using different 3DP technologies.

## 1. Introduction

Three-dimensional printing (3DP) is a disruptive additive manufacturing technology that enables the fabrication of physical objects in a layer-by-layer manner using computer-aided design (CAD) software [1]. Following the US Food and Drug Administration’s (FDA) approval of the first 3D printed tablet for the treatment of epilepsy in children as young as four years old in 2016, Spritam^®^ [2], 3DP is emerging as a novel method of producing medicines by offering a small-batch, highly flexible and rapid manufacture of pharmaceutical products at the point of dispensing [3,4]. 3DP is forecasted to become a revolutionary technology in reshaping the way medicines are manufactured today, evolving from a “one size fits all” approach towards personalized medicines [5].

3DP is an umbrella term that encompasses a variety of different printing technologies [6]. Fused deposition modelling (FDM) is the most widely used 3DP technology in pharmaceutics, owing to its cost-effectiveness and ease of operation. In FDM, thermoplastic filament is used as the feedstock material, made of a range of pharmaceutical grade excipients to fabricate various oral formulations [7,8,9,10,11]. Semi-solid extrusion (SSE), also known as gel extrusion, involves the extrusion of a gel or paste that rapidly solidifies, acting as a support for the next deposited layer [6,12]. Digital light processing (DLP) uses spatially controlled photopolymerization to create solid objects from a photoreactive liquid resin upon light irradiation and has been explored as a result of its high accuracy and superior resolution [13,14]. Selective laser sintering (SLS) is an alternative technology that utilises a laser beam to selectively sinter powder particles and produce the desired object [15]. Adjusting the laser speed can alter the formulation porosity, allowing an extensive range of dosage forms to be fabricated, including orally disintegrating tablets (ODT’s) [16,17] and modified release oral dosage forms [18,19].

The flexibility and personalisation of 3DP offer numerous opportunities, especially in the development of medicines for the paediatric population [20]. This highly diverse population subset, ranging from pre-term neonates to adolescents, presents unique pharmaceutical development challenges, including those related to dose precision, patient acceptability and safety of excipients. Oral administration remains the most convenient route for patients, although they can present limitations related to dose modification, ease of swallowing, taste masking, physical and chemical stability and packaging [21]. Due to the lack of appropriate dosage forms, existing products are often manipulated (e.g., crushing or tablet splitting) or formulations are extemporaneously prepared [22], which may compromise the safety and efficacy of the treatment [23]. Although oral liquids such as syrups are highly acceptable (if palatable) and easy to administer, their benefits are counterbalanced by a risk of dosing error, instability, safety of excipients and storage concerns [24,25].

The need for paediatric-appropriate oral formulations with flexible dosing and easy administration has led to the development of novel 3D printed formulations such as minitablets and chewable dosage forms. Previously, FDM 3DP was used to prepare baclofen-loaded minicaplets [23] and indomethacin-loaded paediatric dosage forms with a Starmix^®^ design demonstrating suitable taste masking properties [26]. Furthermore, paediatric-friendly chewable formulations were also developed using extrusion-based 3D printing, including Lego™-like tablets and chocolate-based dosage forms, overcoming the issue of swallowability and hence improving treatment adherence in children [24,27]. Goyanes et al. reported, for the first time, the manufacture of isoleucine (for the treatment of a rare metabolic disease) into chewable formulations with SSE 3DP in a clinical setting, allowing the small-batch production of medicines at the point of dispensing [4]. This was the first and only study so far that successfully probed the use of 3D printing in a hospital setting to prepare treatments at the dispensing point.

As the opportunities for developing 3D printed paediatric medicines using different technologies continues to grow, it is important to determine end-user perceptions and attitudes towards this novel manufacturing approach. Engaging with prospective patients, their caregivers and healthcare professionals pose several advantages; identifying opportunities for the technology application and improving product design to improve acceptance and uptake [28]. In a recent study, perceptions of healthcare professionals on 3DP pharmaceuticals for paediatrics were investigated, revealing numerous opportunities such as on-demand manufacture, customised doses, child-friendly shapes and the production of small sized dosage forms [29]. The European Medicines Agency (EMA) highlighted the importance of evaluating patient acceptability, namely the ability and willingness to take ones dosage form, preferably with children themselves, as an integral part of the product development process given the significant impact this has on patient adherence and therapeutic outcomes [30]. As children seek greater responsibility over their own therapy as they age, regulatory agencies advise acceptability studies of pharmaceutical products to be assessed with children and not just their caregivers, with evidence displaying more accurate and reliable data from end-users directly [31,32]. Historically, children’s viewpoints have been neglected in clinical research, despite strong evidence that it can have numerous advantages for both the researcher and participants. Indeed, patient acceptability is a critical factor when defining the quality target product profile (QTPP) for medicines intended for paediatric use. QTPP is an integral part of the drug development timeline, a summary of critical product characteristics required to achieve optimal quality, with considerations including route of administration, dosage form, dosage strength and other criteria [33].

Most of the studies on 3DP of paediatric medicines evaluate the use of a 3DP technology to prepare formulations and characterize them in vitro based on the constituent materials, but the reports evaluating user opinions although critical are scarce [29,34,35]. Herein, the aim was to investigate the influence of the initial visual appearance of the printlets prepared using different 3DP technologies on paediatric end-user visual preference, and to evaluate their opinions about the type of solid dosage forms. To the authors’ knowledge, this is the first study investigating the effect of the characteristics of 3D printed tablets with children.

## 2. Materials and Methods

### 2.1. Materials

Polyvinyl alcohol (PVA) filaments were obtained from eSUN (Shenzhen, China). Polyethylene glycol diacrylate (PEGDA, average MW 575 g/mol), bis (2,4,6-trimethylbenzoyl) phenylphosphineoxide and riboflavin (MW 376.36 g/mol) were purchased from Sigma Aldrich Ltd. (Dorset, UK). Kollicoat IR, a copolymer of polyvinyl alcohol and polyethylene glycol (approx. MW 45,000 Da) was obtained from BASF (Stockport, UK). Candurin^®^ Gold Sheen was purchased from Merck (Dorset, UK). All materials were used as received.

### 2.2. Preparation of the 3D Printed Placebo Printlets

The 3D model of the placebo printlets was a cylinder (10 mm diameter × 3.6 mm height) designed with 123D design (Autodesk Inc., San Rafael, CA, USA) and exported as a stereolithography file (.stl) into the 3D printer software. The cylinder shape was chosen as examples of traditional formulations. The .stl format contains only the object surface data, and all the other parameters need to be defined from the corresponding printer software in order to print the desired object.

Four types of printlets were prepared using different 3DP technologies; fused deposition modelling (FDM), digital light processing (DLP), selective laser sintering (SLS) and semi-solid extrusion (SSE).

#### 2.2.1. DLP 3D Printing

The photopolymer solutions were prepared with 1% (*w*/*w*) of bis (2,4,6-trimethylbenzoyl) phenylphosphineoxide as the photoinitiator, 98.9% (*w*/*w*) PEGDA as the photoreactive monomer and 0.1% (*w*/*w*) riboflavin to a total mass of 10 g. The solution was mixed thoroughly for 3 h at room temperature until all the components were fully dissolved, followed by pouring into the resin tray for printing. All placebo printlets were fabricated using a commercial DLP 3D printer (Titan2 HR, Kudo3D Inc., Dublin, CA, USA), equipped with a HD DLP projector, which has a visible light source. The printer XY resolution was set as 23 μm. The .stl file was opened in the Kudo3D Print Job Software (Kudo3D Inc., Dublin, CA, USA) for slicing into layers of 100 µm thickness and sent for printing. The exposure time was selected as 20 s per layer. After printing, all the printlets were post washed with isopropyl alcohol for 20 min and post cured for 20 min at 45 °C in Form Cure (Formlabs Inc., Somerville, MA, USA).

#### 2.2.2. SLS 3D Printing

One hundred grams of a mixture of polymer (Kollicoat IR) and colourant were blended in a pestle and mortar. Three percent of Candurin^®^ Gold Sheen was added as an absorbent, to enhance laser energy absorption and ensure successful printability. The powder mixture was transferred to the SLS printer (Sintratec Kit, AG, Brugg, Switzerland) to formulate the cylindrical printlets (10 mm diameter × 3.6 mm height), which were designed using Autodesk 123D Design (Autodesk). The 3D models were exported as a stereolithography (.stl) file into the Sintratec central software Version 1.1.13. A chamber temperature of 90 °C and a surface temperature of 110 °C were selected. The printing process began with the activation of a 2.3 W blue diode laser (445 nm) (laser scanning speed of 90 mm/s) to sinter the powder on the building platform. The printlets were then removed from the powder bed and any excess powder was brushed off.

#### 2.2.3. Semisolid Extrusion 3D Printing–Chewable Printlets

Chewable placebo printlets were prepared as in a previous study [4]. Excipients used to prepare the chewable formulations (sucrose, pectin, maltodextrin, water and flavourings) were loaded in a syringe and printed using a specially adapted 3D printer, MEDIMAKER™ (FabRx, Kent, UK). Syringes were heated to 70 °C inside the printer to obtain a viscosity suitable for manufacturing the printlets during extrusion.

#### 2.2.4. FDM 3D Printing

The placebo printlets were fabricated with commercial PVA filament, using a standard FDM 3D printer (MakerBot Replicator 2X, MakerBot Inc., Brooklyn, NY, USA). The design .stl file was opened in the 3D printer software (Makerware v3.10.0.1725, MakerBot Inc., Brooklyn, NY, USA). The print settings were defined as follows: infill percentage 50%, number of shells (2), standard resolution (layer height 0.20mm) with no raft, printing temperature 190 °C, speed while extruding 90 mm/s and speed while travelling 150 mm/s.

### 2.3. Visual Preference Semi-Structured Survey with Participants

The survey was completed as a single-site study at an East London primary school with participants aged 4 to 11 years old. Ethical approval was granted by the UCL Research Ethics Committee (REC 4612/026). All school pupils were provided with two age-adapted information sheets about the study, for adults and children, respectively, as well as a consent form for parents/caregivers to complete for their child to be eligible to take part. On the day of the study, a member of the research team explained the study to the child participant and their informed assent was also collected electronically. A short and simple questionnaire was designed electronically (Qualtrics, Provo, UT, USA) to cater to the comprehension and capabilities of the participants.

In the first part of the semi-structured interview, each participant (*n* = 368) was shown the 4 printlets in a clear sealed container and asked to record which printlet they liked (1) the best and (2) the worst by dragging the picture of the printlet in the designated box of the electronic questionnaire. Participants were then asked why they preferred or disliked the printlets and recorded their non-compulsory open responses themselves or with the aid of the researcher. Before the end of the survey, participants were told that printlet no. 3 (SSE) was in fact chewable and the rest were to be swallowed whole. Participants were asked whether this would change their preferences, and responses were added to the comments section of the electronic survey.

In the second part, each participant (*n* = 310) answered open questions or provided comments about the printlets. For the analysis of the results, two researchers independently reviewed open comments and assigned whether they were positive or negative. The comments were reviewed, and four key themes were identified that impacted participant choice—appearance, perceived taste, texture and familiarity (any prior experience with tablets).

### 2.4. Statistical Analysis

Participant details including age and sex and the questionnaire responses were exported into Microsoft Excel 2020 for generating data graphs. IBM SPSS Statistics software version 27.0 (SPSS Inc., Chicago, IL, USA) was employed to perform nonparametric statistical analysis. The Kruskal-Wallis test with pairwise comparison was used to evaluate the significance between the visual preference of each type of printlet. The type of printlet was set as the independent variable and the participant responses were set as the dependent variable with the choice for the best and the worst printlets coded as ‘1′ and ‘−1′ and with the non-selected printlets coded as ‘0′. Chi-square test of homogeneity ($\chi^{2}$) was used for statistical comparisons of the influence of age and gender differences on the visual preferences. The change on the visual preferences of the printlets before and after the participants were told that the SSE printlet was a chewable dosage form were also studied using the Chi-square test. The data collected from the survey were recorded as the observed values and the expected values were calculated accordingly. A *p*-value less than 0.05 was considered statistically significant throughout the analysis.

## 3. Results

### 3.1. Printlet Preparation

**Figure 1:**
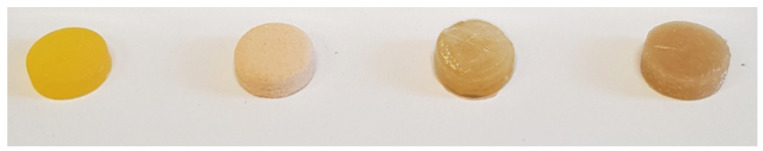
Placebo printlets fabricated with the four different 3DP technologies. From left to right: digital light processing (DLP), selective laser sintering (SLS), semi-solid extrusion (SSE) and fused deposition modelling (FDM).

The selected 3D design of all placebo printlets was a cylinder or ‘disc’ to match the appearance of conventional formulations. In a previous acceptability study on 3D-printed dosage forms, the ‘disc’ shape was evaluated as being easier to pick and swallow by adult participants in comparison with other shapes [35]. Placebo printlets were prepared using four different 3DP technologies; DLP, SLS, SSE and FDM (Figure 1). All the printlets showed similar dimensions and appearance, displaying the consistent reproducibility that 3DP offers.

Due to the original colour of the commercial PVA filament used in this study, the FDM printlets appeared yellow in colour (Figure 1). In order to match the colour with the other formulations prepared with the other 3D printing technologies, colourants were added to some placebo printlets. Riboflavin was used in DLP and Candurin^®^ Gold Sheen in SLS. The DLP printlets had a smooth surface finish and showed a significantly brighter yellow colour than the others. This was a result of the incorporation of 0.1% (*w*/*w*) riboflavin in the formulation. In comparison to the others, DLP printlets had well-defined and smooth edges, owing to the technology’s high resolution. On the other hand, the surface of the SLS printlets was rough and crumbly, characteristic of the powder bed fusion-based technology since no solvent binder is used in the process. SLS 3DP does not melt the powder particles but only sinters them together, resulting in the lightest colour printlets. SSE printlets showed a lower resolution, which is evident in the final print, with visible patterns of the individual layers (Figure 1). Although they have a rugged surface compared to the rest of the printlets, this is not an inconvenience for swallowing since they are chewable formulations with a softer and gummy-like texture. In comparison to other 3DP techniques, the SSE printlets also appear to be shinier in appearance, owing to the use of food-grade ingredients in the placebo formulations. Out of all extrusion-based 3DP technologies (FDM and SSE), FDM has the best resolution, which is evident from the even surface and cohesion of all the deposited layers. However, DLP and SLS show better resolution than the extrusion-based 3DP technologies.

### 3.2. Visual Preference of Printlets

**Figure 2:**
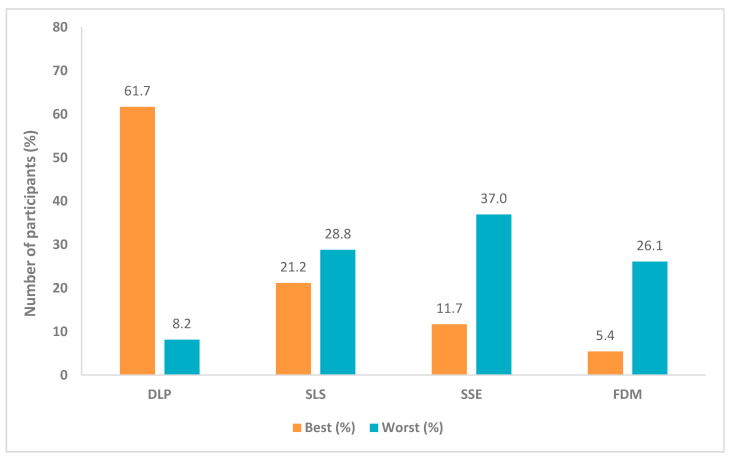
Printlet visual preference results summary (*n* = 368).

A total of 368 children (aged 4–11 years) took part in the first part of the survey by selecting which they considered to be the visually best and the worst printlets. Of these, 310 participants recorded further responses in the individual open questionnaire regarding the visual printlet descriptions on appearance, perceived taste, texture and familiarity. Overall, the DLP printlet was initially the most preferred, with 61.7% (*n* = 227) of children rating it as the best (Figure 2), and only 8.2% (*n* = 30) selecting it as the worst. In comparison, the FDM printlets were chosen as favourite by the least number of participants (5.4%, *n* = 20) and the SSE chewable printlets were chosen as the worst visually by the largest number of children (37.0%, *n* = 136), making these two printlets the least visually appealing for paediatrics. Statistical analysis revealed that the visual preferences of the printlets were statistically different (*p* < 0.001), however the pairwise comparison results indicated that there were no significant differences between the SSE printlets and FDM printlets (*p* > 0.05).

**Figure 3:**
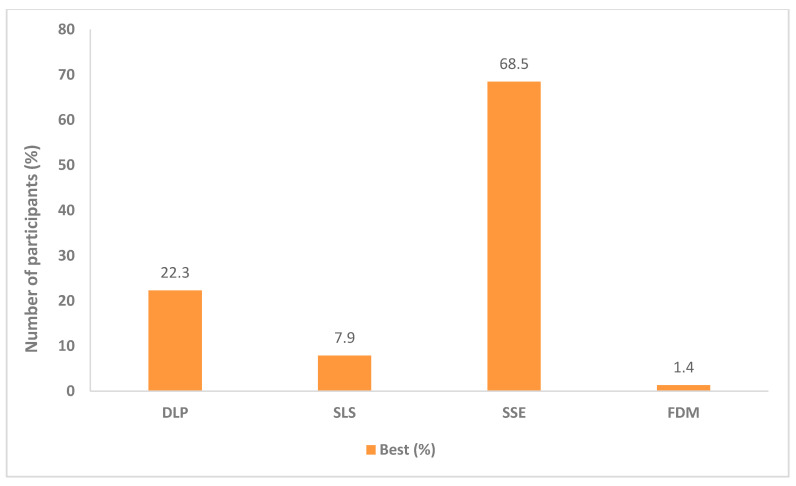
Printlet visual preference results summary after the participants knew the SSE printlet is a chewable form (*n* = 368).

After submitting their choices, the participants were told that the SSE printlets were in a chewable form like gummies and were given the opportunity to change their preferences if they wanted to. Out of a total of 310 participants who completed the voluntary open questionnaire, 79% (*n* = 245) of the children said that they would now choose the SSE printlets as their favourite regardless of their original choice. SSE printlets now chosen as the best rose from 11.7% (*n* = 43) to 68.5% (*n* = 252) and the preferences for the other printlets were significantly lower (Figure 3). Among the 21% (*n* = 65) of participants who stayed with their original choice, some said that this is only because they like/prefer swallowing tablets as the administration process is faster compared to chewing, especially if the chewable form exhibits an unpleasant taste. The chi-square test showed that there were significant differences in visual preference for the selection of best printlets before and after the children were informed that the SSE printlets were chewable dosage forms ($\chi^{2}$(3) = 247.55, *p* < 0.001).

### 3.3. Visual Preference Survey Results Based on Age and Sex

**Figure 4:**
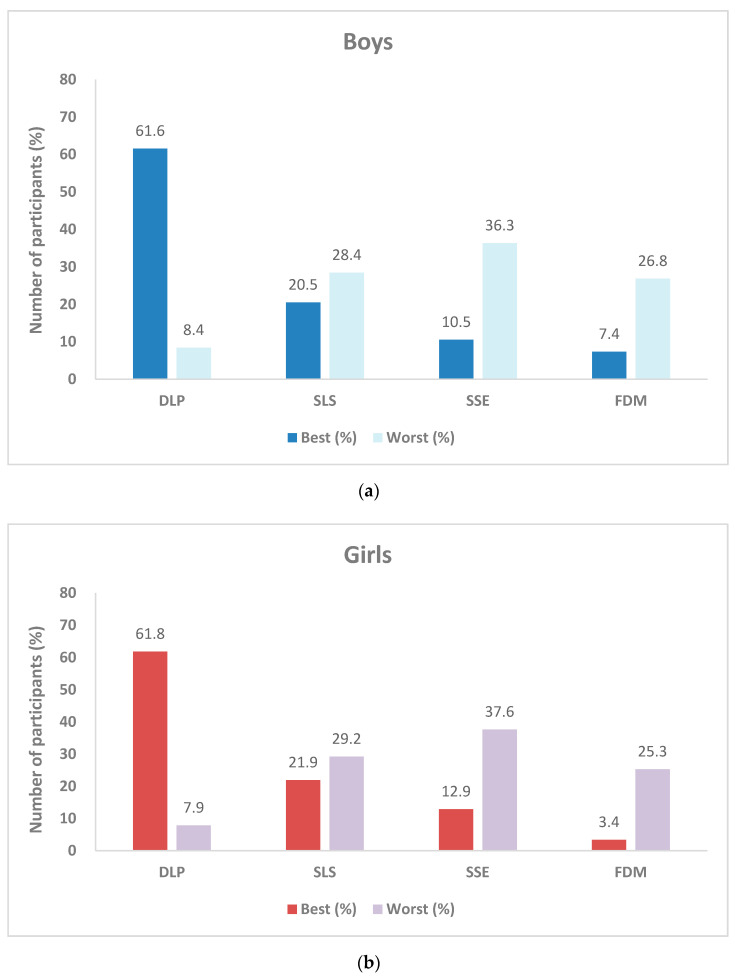
Printlet visual preference results based on sex: (**a**) Boys and (**b**) girls.

Of the 368 children recruited in the study, the participant sex was divided as 190 boys (51.6%) and 178 girls (48.4%). The same trend was observed in boys and girls regarding the preference for the best printlets (from best to worst); DLP, SLS, SSE, FDM and for the worst printlets (from worst to best); SSE, SLS, FDM, DLP (Figure 4). For the DLP, SLS and SSE printlets, the visual preference for boys and girls was highly similar on their choices of the best and worst. The chi-square test results showed that there were no differences in visual preference between boys and girls for the choice of best printlets ($\chi^{2}$(3) = 3.24, *p* > 0.05) and for the choice of worst printlets ($\chi^{2}$(3) = 0.18, *p* > 0.05). Overall, it was found that gender does not have a significant effect on the visual preference of printlets, and that the same 3DP oral dosage forms can be given to both boys and girls with similar preferences.

**Figure 5:**
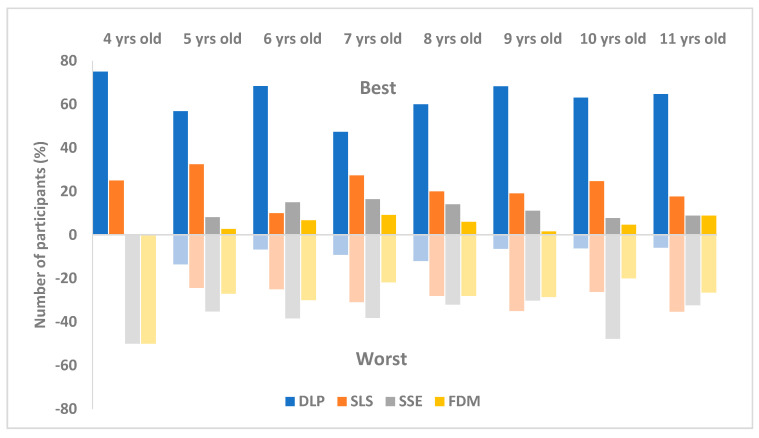
Printlet visual preference results based on different age groups (*n* = 368).

Further analysis was carried out on the results based on age (Figure 5). The participants covered a range of eight age groups: 4 years old (*n* = 4), 5 years old (*n* = 37), 6 years old (*n* = 60), 7 years old (*n* = 55), 8 years old (*n* = 50), 9 years old (*n* = 63), 10 years old (*n* = 65) and 11 years old (*n* = 34). As can be seen from the results in Figure 4, the DLP printlets received the most votes for being the visually best for all age groups, followed by the SLS and SSE printlets as the second and the third best except for the six-year-old group. Regarding the opinion on the visually worst printlets, most of the age groups showed a similar trend on their preferences in the following order (from best to worst); DLP, SLS, FDM, SSE. In the 9-year-old and 11-year-old groups, the SSE printlets were slightly preferred over the SLS printlets. Statistical analysis results showed that there were no differences in visual preference among different age groups for the choice of best printlets ($\chi^{2}$(21) = 19.18, *p* > 0.05) and for the choice of worst printlets ($\chi^{2}$(21) = 13.21, *p* > 0.05). The present study suggested that age was not found to significantly impact the visual preference of printlets.

### 3.4. Visual Printlet Descriptions

The participants were asked why they preferred or disliked certain printlets and their comments and descriptions of each were recorded. A total of 310 participants voluntarily completed the survey themselves or with the aid of the researcher (response rate 84.2%).

**Figure 6:**
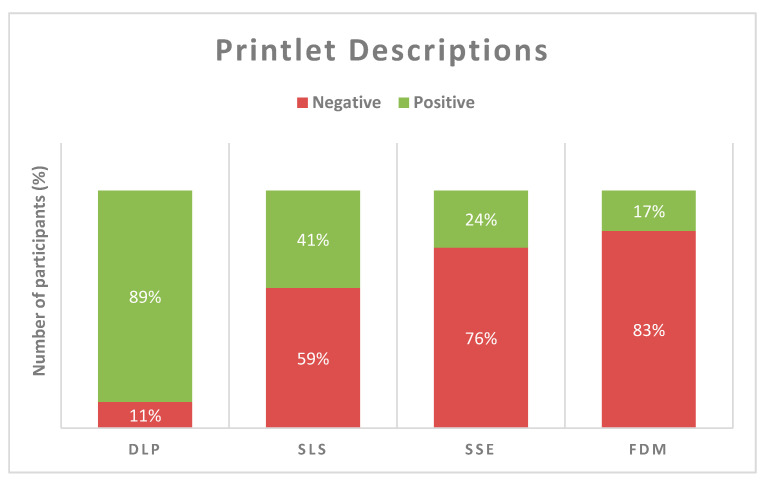
Overall visual description data for all printlets based on the selected 3D printing (3DP) technology (DLP *n* = 244; SLS *n* = 170; SSE *n* = 125; FDM *n* = 92).

Of these comments (*n* = 651), the DLP printlets received the highest positive feedback of 89% (*n* = 217), while the other three printlets received a considerably lower proportion (Figure 6). Amongst the printlets, the FDM printlets received the lowest popularity from the children, with the highest negative feedback of 83% (*n* = 76).

**Figure 7:**
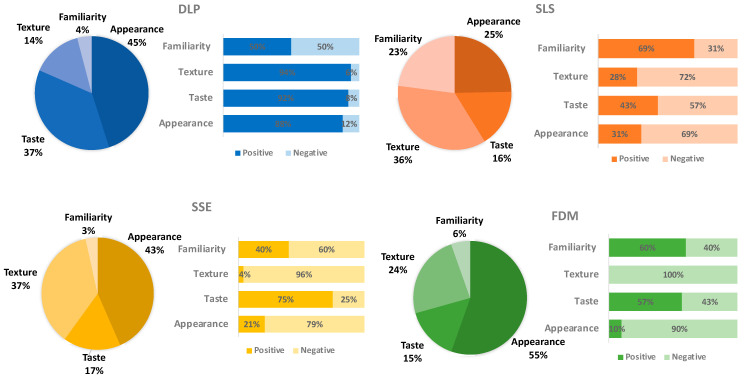
Summary of visual description data for printlets manufactured by different 3DP technologies based on four categories: Appearance, perceived taste, texture and familiarity (DLP *n* = 244, SLS *n* = 170, SSE *n* = 125, FDM *n* = 92). Each pie chart shows the percentage of comments given for each printlet. The four categories of each pie chart are split into ‘positive’ and ‘negative’ comments, which correspond to each 3DP technology, with positive comments represented by darker shades and negative comments by lighter shades.

The visual description data were further analysed for each 3DP technology and grouped into four distinct categories; appearance, perceived taste, texture and familiarity, all factors that affected their printlet choice (Figure 7). Here, the category of ‘appearance’ has included any descriptions mentioning the shape, size or colour of the printlets. The ‘perceived taste’ category consists of opinions on how the printlets would taste if taken, while the ‘texture’ category includes comments describing the visual texture of the printlet surface. ‘Familiarity’ refers to any comments given based on experience with actual tablets, either taken themselves or by family members.

Of the comments given to the DLP printlets, appearance, taste and texture received higher proportions of positive comments over negative ones, unlike familiarity, which had the same number of both. As a result, visual appearance had the greatest effect on the DLP printlets being chosen as the best, owing to nearly half of all the comments (45%, *n* = 110), with the majority saying that the DLP printlets had a ‘nice colour’, were ‘shiny’ or ‘see-through’. Appearance was closely followed by perceived taste (37%, *n* = 89) as many children mentioned that the printlets looked like a gummy/sweet or that they would taste like a lemon/orange. These two categories are closely related as the bright yellow colour of the printlets may lead to the belief that they are lemon-flavoured sweets. A large proportion of children described the texture of the DLP printlets as ‘smooth’ and therefore easier to swallow by patients, which was attributed to the high resolution of the DLP technology. Although not many comments were given based on familiarity (14%, *n* = 10), half of the negative descriptions comprised of adjectives such as ‘weird’, ‘plastic’, ‘fake’, ‘rubber like’ and the fact that it does not look like a real tablet. Children are used to being given simple white tablets, which are manufactured using compression techniques, therefore they may be sceptical of brightly coloured ones, assuming they are not medicine.

Many of the comments to describe the SLS printlets were based on texture (36%, *n* = 61) and appearance (25%, *n* = 42), with many children describing the printlets negatively as ‘powdery/crumbly’, ‘dry’, ‘sandy’ and like a ‘sponge’. In comparison to the rest of the 3DP technologies, SLS is the only one that utilises powder alone, resulting in printlets that are powdery and fragile, which seemed to be a visual characteristic that was not favoured by children. As a result, these printlets received only one comment on the similarity to a sweet in the perceived taste category. On the contrary, positive paediatric feedback on the SLS printlets derived from previous experience and familiarity with tablets that appeared like the printlets. Sixty-nine percent (*n* = 27) of all the familiarity comments were positive, either because the participants have had similar tablets in the past or that the printlets were like real tablets. Eleven participants mentioned that the SLS printlets looked like they would dissolve quicker, another reason why they were their favourite. However, some children mentioned prior use of “dissolving” tablets that were unpalatable, therefore making them rate these printlets as the worst choice. SLS also received a high proportion of negative comments in the perceived taste category (57%, *n* = 16), because many children assumed the printlets would taste bitter/horrible as they dissolve.

Even though 79% (*n* = 245) of participants chose the SSE printlets as their favourite once knowing they were chewable, they were not their favourite originally, mostly as a result of visual characteristics. Appearance (43%, *n* = 63) and texture (37%, *n* = 53) were important attributes for the children when describing the SSE printlets. 79% (*n* = 50) of the participants used negative adjectives to describe the visual appearance of the printlets, including ‘broken’, ‘weird’, ‘mouldy’, ‘not a nice colour’ and ‘dirty’. The printlets had to be of a similar colour to the rest, resulting in a pale-yellow appearance, which the children did not like. In contrast to DLP 3DP, the resolution of SSE is the lowest out of all the technologies [3], evident in 96% (*n* = 51) of the texture comments being ‘rough’, ‘slimy’ and ‘bumpy/lumpy’. Out of all four categories, perceived taste alone had the majority of positive comments (75%, *n* = 18) largely due to the fact that many children believed that they looked like gummies/sweets and would taste nice, again proving the great importance of taste over the other three categories on paediatric printlet choice.

The FDM printlets were the least favoured by the participants, receiving the lowest number of comments (*n* = 92) and the lowest proportion of positive feedback (17%, *n* = 16). More than half of the comments were given based on appearance (55%, *n* = 51), however 90% (*n* = 46) of them were negative with the most commonly used adjectives being ‘mouldy’, ‘weird’, ‘dirty’ and ‘old’, owing to the dark colour of the filament used to prepare the printlets. The FDM printlets were the only ones to receive 100% (*n* = 22) negative feedback in the texture category, with many children describing the printlets as ‘rough’, ‘sticky’ and ‘hard’. Again this may be due to the limited printing resolution of extrusion-based 3DP technologies [3]. From the 57% (*n* = 8) of positive taste feedback, most of the results were from the fact that some children perceived the FDM printlets to be sweets/gummies/caramel like the SSE printlets or that they looked nice. Only five comments in total were given on familiarity, and two out of five children indicated that the FDM printlets looked like real tablets.

## 4. Discussion

### 4.1. Visual Preference of Printlets

Presently, the children were not asked if they would be able or willing to take these printlets, but which one they thought looked the best and the worst. Palatability is a key driver for acceptability and the first palatability encounter with the printlet is what one sees, followed by orthonasal smell and then only buccal taste modalities if consumed. Therefore, the visual preference survey is important as a preamble of acceptability.

Overall, the DLP printlets received the highest popularity in both boys and girls regardless of different age groups, owing to its bright colour, superior resolution and smooth surface finish. Recently, multiple pharmaceutical applications have been reported utilising these vat photopolymerization 3DP technologies such as SLA and DLP, including drug-loaded oral dosage forms [35,36,37] and hydrogels [38,39], polypills [40,41], as well as patient-specific medical devices [42,43,44]. However, the lack of generally recognised as safe (GRAS) photocurable materials remains a challenge. Although numbers of biocompatible dental resins are now available on the market [45,46], they are not intended or approved for biomedical applications, especially in the human body [47]. Herein, it is expected that novel and safe photoreactive biomaterials could be developed for 3D printing for drug delivery purposes. However, selecting excipients for use in paediatric products requires additional special safety considerations. Usually, a more conservative approach is favoured in the case of limited safety data as excipients can result in different exposure levels in children of different ages [30].

From the results of this study, the SLS printlets were considered the second-favoured overall amongst the children. This result suggests that children would prefer oral dosage forms that disperse or dissolve in the mouth, such as orodispersible tablets (ODTs) or chewable formulations instead of having to swallow whole tablets. This was proven in a recent study analysing children’s preferences and demonstrating that children in the UK preferred ODTs over tablets, capsules and liquid dosage forms [48].The SLS process poses several advantages over other existing 3DP technologies, being a single step and solvent-free approach to medicine manufacture, having the capability of printing intricate and complex structures due to the precision of the laser [49,50]. Similar to the Zipdose^®^ technology that is used for Spritam^®^ [2], SLS is also a promising technology to fabricate ODTs, which can rapidly disintegrate within 4 s [17]; however, no in vivo studies have been conducted so far. Additionally, some children indicated that they were worried about the taste of ODTs in their mouth, confirming that taste masking is an important aspect of paediatric formulation development, especially for ODTs with aversive APIs [51].

Recent advancements have been made with the SSE technology, including formulating orodispersible films (ODFs) [52], multiple drug-loaded tablets [53] and immediate release dosage forms with high drug loads [54]. Although the initial perception about the SSE printlets was not desirable compared to other printlets, most of the children changed their minds once they were aware that the SSE printlets were a chewable formulation, highlighting the popularity of chewable tablets in the paediatric population. This was also evidenced in the study conducted by Ranmal et al. where chewable medicines were preferred over orodispersible and multiparticulate formulations amongst school children and adolescents [32]. Although some children might find it unpleasant with the residue taste of the chewable formulation, this could be overcome by the addition of fruit flavoured agents [55].

The SSE technology allows the addition of various colourants or food dyes based on the patient’s preference, reducing the hurdle of low patient compliance [4,27]. In a recent clinical study on manufacturing isoleucine SSE printlets as a treatment for children with Maple Syrup Urine Disease (MSUD) conducted in a hospital setting, the results showed that although all the printlets were well accepted, the strawberry (red), orange (orange) and lemon (yellow) flavoured printlets were preferred over the other flavours offered: Coconut (black), banana (light green) and raspberry (light blue) others [4]. This together with the finding from this study indicates that children show higher preference towards brightly coloured medicines. The choice of suitable excipients in a paediatric medicine remains one of the key elements of its pharmaceutical development. Therefore, it is important to assess and justify the safety risk versus the potential benefit before adding colouring agents or flavourings [30].

On the other hand, familiarity was found to affect the children’s perception in this study. Although the printlet shapes were the same, children showed positive perception towards the SLS printlets, attributing to their familiarity of the texture. This finding was in agreement with a previous study where patients showed higher preference for capsule- and disc-shaped printlets, which have similar appearance to conventional formulations [35].

FDM is the most widely used 3D printing technology in pharmaceutics, owing to its cost-effectiveness and easy operation and has been shown to have good patient acceptability [35]. Moreover, a range of pharmaceutical grade polymers and excipients can be used for this technology to prepare oral dosage forms with unique or personalised release profiles [9,56]. For the preparation of the FDM printlets, a water-soluble PVA filament was used. PVA is a commonly used polymer in pharmaceutics, owing to its high stability, safety and chemical inertness [57]. As a result of its mechanical properties, PVA has been also used extensively as a drug carrier in 3DP pharmaceutical formulations [58]. However, this study shows that visual preference of FDM printlets amongst children (aged 4–11 years) was relatively low compared to the other printlets. This was mostly attributed to their hard appearance and rough texture, features that have been found to be unpleasant for children in oral dosage forms.

### 4.2. Printlet Characteristics for a Quality Target Product Profile (QTPP)

In order to select the best formulation for the paediatric population, different 3DP technology printlet characteristics must be compared to create an effective quality target product profile (QTPP) (Table 1). Both knowledge of the 3DP technologies and user opinion data are critical in establishing key quality attributes for the paediatric population, a patient group that has been neglected in acceptability studies in the past. From the desirable dosage form features that were highlighted by the children in the study, it is clear from Table 1 that all four 3DP technologies can manufacture flexible solid oral dosage forms, alongside easy manipulation of colour and taste that today’s mass manufacturing techniques cannot accomplish. Although the DLP printlets were the most visually appealing to children as a result of the bright colour and smooth texture, there are no data in the current literature to prove that this 3DP technology can be used to manufacture chewable or orodispersible formulations, and there are currently no pharmaceutical grade excipients that can be used with this technology. Both SLS and FDM are very similar in their capabilities of producing child- friendly formulations. The participants highlighted the importance of a smooth surface and acceptable texture, features that require improvement in both technologies as a result of a lower resolution in FDM and a powder finish in SLS. However, both 3DP techniques are suitable for safe pharmaceutical manufacture, capable of producing orodispersible formulations that have also been found to be favourable amongst the participants.

Based on the visual preference study carried out and the capabilities of each 3DP technique, it is evident that SSE shows the most potential for manufacturing solid oral dosage forms that will meet the specific requirements of the paediatric population (Table 1). This technology is to date the only 3DP technology capable of producing chewable formulations, a factor that currently seems to be highly favourable when developing paediatric pharmaceutical products.

## 5. Conclusions

Four types of printlets were prepared using four different 3DP technologies, DLP, SLS, SSE and FDM. The children selected DLP printlets as their visually favourite, obtaining the highest results in our visual preference survey based on the four categories: Appearance, perceived taste, texture and familiarity. However, once the participants were told that the SSE printlets were a chewable dosage form, many children chose these printlets as their favourite over their original choice, no matter the appearance described initially. FDM printlets were chosen as the least favourite by the children, with the lowest scores obtained. No significant visual preference differences were found regarding participant age and sex. Although appearance was significant in identifying the participants best and worst printlet type, other factors such as perceived taste, texture and familiarity showed varied results for each printlet type. The SLS and FDM printlets were less well received but obtained positive comments for looking familiar to participants who had taken similar looking formulations in the past (ODTs or tablets). It was found that although visual appearance (colour, shape) of printlets play a key part in a child’s first impression, the preferred dosage form will still be chosen by the child if they do not have to swallow the printlet intact, which should have a nice taste; two vital characteristics that need to be considered in paediatric-centred formulation development of 3DP dosage forms. This is the first study to evaluate children’s perceptions of printlets manufactured by four different 3DP techniques, and the findings are beneficial for further research on paediatric formulation development using 3DP.

## Figures and Tables

**Table 1:** A summary of the key attributes for the development of 3D printed paediatric formulations.

Feature	DLP	SLS	SSE	FDM
Age-appropriate formulation design (different sizes and shapes)	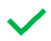	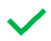	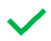	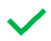
Manufacture of multiparticulate systems	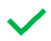	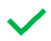	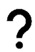	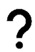
Manufacture of chewable formulations	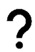	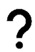	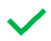	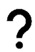
Manufacture of dispersible formulations	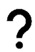	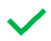	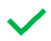	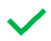
Use of pharmaceutical grade excipients with an established safety profile in the target paediatric age group(s)	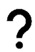	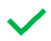	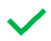	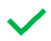
Taste masking capabilities through the addition of sweeteners/flavourings	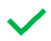	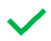	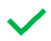	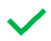
Selection of colour	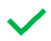	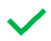	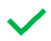	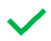
Aesthetically smooth surface	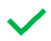	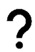	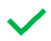	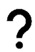

## References

[1] Basit A.W., Gaisford S. (2018). 3d Printing of Pharmaceuticals.

[2] Aprecia Pharmaceuticals Fda Approves the First 3d Printed Drug Product. https://www.aprecia.com/news/fda-approves-the-first-3d-printed-drug-product.

[3] Awad A., Trenfield S.J., Goyanes A., Gaisford S., Basit A.W. (2018). Reshaping drug development using 3d printing. Drug Discov. Today.

[4] Goyanes A., Madla C.M., Umerji A., Piñeiro G.D., Montero J.M.G., Diaz M.J.L., Barcia M.G., Taherali F., Sánchez-Pintos P., Couce M.-L. (2019). Automated therapy preparation of isoleucine formulations using 3d printing for the treatment of msud: First single-centre, prospective, crossover study in patients. Int. J. Pharm..

[5] Trenfield S.J., Awad A., Madla C.M., Hatton G.B., Firth J., Goyanes A., Gaisford S., Basit A.W. (2019). Shaping the future: Recent advances of 3d printing in drug delivery and healthcare. Expert Opin. Drug Deliv..

[6] Vithani K., Goyanes A., Jannin V., Basit A., Gaisford S., Boyd B. (2019). An overview of 3d printing technologies for soft materials and potential opportunities for lipid-based drug delivery systems. Pharm. Res..

[7] Trenfield S.J., Xian Tan H., Awad A., Buanz A., Gaisford S., Basit A.W., Goyanes A. (2019). Track-and-trace: Novel anti-counterfeit measures for 3D printed personalized drug products using smart material inks. Int. J. Pharm..

[8] Sadia M., Sośnicka A., Arafat B., Isreb A., Ahmed W., Kelarakis A., Alhnan M.A. (2016). Adaptation of pharmaceutical excipients to fdm 3d printing for the fabrication of patient-tailored immediate release tablets. Int. J. Pharm..

[9] Melocchi A., Parietti F., Maroni A., Foppoli A., Gazzaniga A., Zema L. (2016). Hot-melt extruded filaments based on pharmaceutical grade polymers for 3d printing by fused deposition modeling. Int. J. Pharm..

[10] Elbadawi M., Castro B.M., Gavins F.K., Ong J.J., Gaisford S., Pérez G., Basit A.W., Cabalar P., Goyanes Á. (2020). M3diseen: A novel machine learning approach for predicting the 3d printability of medicines. Int. J. Pharm..

[11] Fina F., Goyanes A., Rowland M., Gaisford S., W Basit A. (2020). 3d printing of tunable zero-order release printlets. Polymers.

[12] Seoane-Viaño I., Ong J.J., Luzardo-Álvarez A., González-Barcia M., Basit A.W., Otero-Espinar F.J., Goyanes A. (2020). 3d printed tacrolimus suppositories for the treatment of ulcerative colitis. Asian J. Pharm. Sci..

[13] Gardan J. (2016). Additive manufacturing technologies: State of the art and trends. Int. J. Prod. Res..

[14] Xu X., Awad A., Robles-Martinez P., Gaisford S., Goyanes A., Basit A.W. (2020). Vat photopolymerization 3d printing for advanced drug delivery and medical device applications. J. Control. Release.

[15] Awad A., Fina F., Goyanes A., Gaisford S., Basit A.W. (2020). 3d printing: Principles and pharmaceutical applications of selective laser sintering. Int. J. Pharm..

[16] Allahham N., Fina F., Marcuta C., Kraschew L., Mohr W., Gaisford S., Basit A.W., Goyanes A. (2020). Selective laser sintering 3d printing of orally disintegrating printlets containing ondansetron. Pharmaceutics.

[17] Fina F., Madla C.M., Goyanes A., Zhang J., Gaisford S., Basit A.W. (2018). Fabricating 3d printed orally disintegrating printlets using selective laser sintering. Int. J. Pharm..

[18] Awad A., Fina F., Trenfield S.J., Patel P., Goyanes A., Gaisford S., Basit A.W. (2019). 3d printed pellets (miniprintlets): A novel, multi-drug, controlled release platform technology. Pharmaceutics.

[19] Fina F., Goyanes A., Gaisford S., Basit A.W. (2017). Selective laser sintering (sls) 3d printing of medicines. Int. J. Pharm..

[20] Preis M., Öblom H. (2017). 3d-printed drugs for children—Are we ready yet?. AAPS PharmSciTech.

[21] Strickley R.G., Iwata Q., Wu S., Dahl T.C. (2008). Pediatric drugs—A review of commercially available oral formulations. J. Pharm. Sci..

[22] Rautamo M., Kvarnstrom K., Siven M., Airaksinen M., Lahdenne P., Sandler N. (2020). A focus group study about oral drug administration practices at hospital wards-aspects to consider in drug development of age-appropriate formulations for children. Pharmaceutics.

[23] Palekar S., Nukala P.K., Mishra S.M., Kipping T., Patel K. (2019). Application of 3d printing technology and quality by design approach for development of age-appropriate pediatric formulation of baclofen. Int. J. Pharm..

[24] Karavasili C., Gkaragkounis A., Moschakis T., Ritzoulis C., Fatouros D.G. (2020). Paediatric-friendly chocolate-based dosage forms for the oral administration of both hydrophilic and lipophilic drugs fabricated with extrusion-based 3d printing. Eur. J. Pharm. Sci..

[25] Trofimiuk M., Wasilewska K., Winnicka K. (2019). How to modify drug release in paediatric dosage forms? Novel technologies and modern approaches with regard to children’s population. Int. J. Mol. Sci.

[26] Scoutaris N., Ross S.A., Douroumis D. (2018). 3d printed “starmix” drug loaded dosage forms for paediatric applications. Pharm. Res..

[27] Rycerz K., Stepien K.A., Czapiewska M., Arafat B.T., Habashy R., Isreb A., Peak M., Alhnan M.A. (2019). Embedded 3d printing of novel bespoke soft dosage form concept for pediatrics. Pharmaceutics.

[28] Pushparajah D.S. (2018). Making patient engagement a reality. Patient.

[29] Rautamo M., Kvarnstrom K., Siven M., Airaksinen M., Lahdenne P., Sandler N. (2020). Benefits and prerequisites associated with the adoption of oral 3d-printed medicines for pediatric patients: A focus group study among healthcare professionals. Pharmaceutics.

[30] Agency E.M. (2013). Guideline on Pharmaceutical Development of Medicines for Paediatric Use.

[31] El-Rachidi S., LaRochelle J.M., Morgan J.A. (2017). Pharmacists and pediatric medication adherence: Bridging the gap. Hosp. Pharm..

[32] Ranmal S.R., Cram A., Tuleu C. (2016). Age-appropriate and acceptable paediatric dosage forms: Insights into end-user perceptions, preferences and practices from the children’s acceptability of oral formulations (calf) study. Int. J. Pharm..

[33] Yu L.X., Amidon G., Khan M.A., Hoag S.W., Polli J., Raju G.K., Woodcock J. (2014). Understanding pharmaceutical quality by design. AAPS J..

[34] Fastø M.M., Genina N., Kaae S., Kälvemark Sporrong S. (2019). Perceptions, preferences and acceptability of patient designed 3d printed medicine by polypharmacy patients: A pilot study. Int. J. Clin. Pharm..

[35] Goyanes A., Scarpa M., Kamlow M., Gaisford S., Basit A.W., Orlu M. (2017). Patient acceptability of 3d printed medicines. Int. J. Pharm..

[36] Wang J., Goyanes A., Gaisford S., Basit A.W. (2016). Stereolithographic (sla) 3d printing of oral modified-release dosage forms. Int. J. Pharm..

[37] Martinez P.R., Goyanes A., Basit A.W., Gaisford S. (2018). Influence of geometry on the drug release profiles of stereolithographic (sla) 3d-printed tablets. AAPS PharmSciTech.

[38] Karakurt I., Aydoğdu A., Çıkrıkcı S., Orozco J., Lin L. (2020). Stereolithography (sla) 3d printing of ascorbic acid loaded hydrogels: A controlled release study. Int. J. Pharm..

[39] Larush L., Kaner I., Fluksman A., Tamsut A., Pawar A.A., Lesnovski P., Benny O., Magdassi S. (2017). 3d printing of responsive hydrogels for drug-delivery systems. J. 3D Print. Med..

[40] Xu X., Robles-Martinez P., Madla C.M., Joubert F., Goyanes A., Basit A.W., Gaisford S. (2020). Stereolithography (sla) 3d printing of an antihypertensive polyprintlet: Case study of an unexpected photopolymer-drug reaction. Addit. Manuf..

[41] Robles-Martinez P., Xu X., Trenfield S.J., Awad A., Goyanes A., Telford R., Basit A.W., Gaisford S. (2019). 3d printing of a multi-layered polypill containing six drugs using a novel stereolithographic method. Pharmaceutics.

[42] Goyanes A., Det-Amornrat U., Wang J., Basit A.W., Gaisford S. (2016). 3d scanning and 3d printing as innovative technologies for fabricating personalized topical drug delivery systems. J. Control. Release.

[43] Vivero-Lopez M., Xu X., Muras A., Otero A., Concheiro A., Gaisford S., Basit A.W., Alvarez-Lorenzo C., Goyanes A. (2020). Anti-biofilm multi drug-loaded 3d printed hearing aids. Mater. Sci. Eng. C.

[44] Lim S.H., Ng J.Y., Kang L. (2017). Three-dimensional printing of a microneedle array on personalized curved surfaces for dual-pronged treatment of trigger finger. Biofabrication.

[45] Formlabs High-Accuracy 3d Printing Materials for Dental Labs and Practices. https://dental.formlabs.com/uk/materials/.

[46] Next Dent. Leading Dental Materials for 3d Printing. https://nextdent.com/.

[47] Formlabs. Are Formlabs Resins Food-Safe or Biocompatible?. https://support.formlabs.com/s/article/Are-Formlabs-resins-food-safe-or-biocompatible?language=en_US.

[48] Alyami H., Dahmash E., Alyami F., Dahmash D., Huynh C., Terry D., Mohammed A.R. (2017). Dosage form preference consultation study in children and young adults: Paving the way for patient-centred and patient-informed dosage form development. Eur. J. Hosp. Pharm..

[49] Fina F., Goyanes A., Madla C.M., Awad A., Trenfield S.J., Kuek J.M., Patel P., Gaisford S., Basit A.W. (2018). 3d printing of drug-loaded gyroid lattices using selective laser sintering. Int. J. Pharm..

[50] Williams J.M., Adewunmi A., Schek R.M., Flanagan C.L., Krebsbach P.H., Feinberg S.E., Hollister S.J., Das S. (2005). Bone tissue engineering using polycaprolactone scaffolds fabricated via selective laser sintering. Biomaterials.

[51] Lopez F.L., Ernest T.B., Tuleu C., Gul M.O. (2015). Formulation approaches to pediatric oral drug delivery: Benefits and limitations of current platforms. Expert Opin. Drug Deliv..

[52] Yan T.T., Lv Z.F., Tian P., Lin M.M., Lin W., Huang S.Y., Chen Y.Z. (2020). Semi-solid extrusion 3d printing odfs: An individual drug delivery system for small scale pharmacy. Drug Dev. Ind. Pharm..

[53] Khaled S.A., Burley J.C., Alexander M.R., Yang J., Roberts C.J. (2015). 3d printing of tablets containing multiple drugs with defined release profiles. Int. J. Pharm..

[54] Khaled S.A., Alexander M.R., Wildman R.D., Wallace M.J., Sharpe S., Yoo J., Roberts C.J. (2018). 3d extrusion printing of high drug loading immediate release paracetamol tablets. Int. J. Pharm..

[55] Mistry P., Batchelor H., SPaeDD-UK Project (2017). Evidence of acceptability of oral paediatric medicines: A review. J. Pharm. Pharmacol..

[56] Öblom H., Zhang J., Pimparade M., Speer I., Preis M., Repka M., Sandler N. (2019). 3d-printed isoniazid tablets for the treatment and prevention of tuberculosis—personalized dosing and drug release. AAPS PharmSciTech.

[57] DeMerlis C.C., Schoneker D.R. (2003). Review of the oral toxicity of polyvinyl alcohol (pva). Food Chem. Toxicol..

[58] Xu X., Zhao J., Wang M., Wang L., Yang J. (2019). 3d printed polyvinyl alcohol tablets with multiple release profiles. Sci. Rep..

